# IT-Enabled Clinical Decision Support: An Empirical Study on Antecedents and Mechanisms

**DOI:** 10.1155/2018/6945498

**Published:** 2018-12-13

**Authors:** Rogier van de Wetering

**Affiliations:** Faculty of Management, Science and Technology, Open University, Heerlen 6401 DL, Netherlands

## Abstract

Modern hospitals increasingly make use of innovations and information technology (IT) to improve workflow and patient's clinical journey. Typical innovative solutions include patient records and clinical decision support systems to enhance the process of decision making by doctors and other healthcare practitioners. However, currently, it remains unclear how hospitals could facilitate and enable such a decision support capability in clinical practice. We ground our work on the resource-based view of the firm and put forth the notion of IT-enabled capabilities which emphasizes critical IT investment and capability development areas that hospitals could exploit in their quest to improve clinical decision support. We develop a research model that explains how “health information exchange” and enhanced “information capability” collectively drive a hospital's “clinical decision support capability.” We used partial least squares path modeling on large-scale cross-sectional data from 720 European hospitals. Outcomes suggest that health information exchange positively impacts information capability. In turn, information capability complementary partially mediates the relationship between information exchange and clinical decision support. Hence, this research contributes to the literature on clinical decision support and provides valuable insights into how to support such innovative technologies and capabilities in clinical practice. We conclude with a discussion and conclusion. Also, we outline the inherent limitations of this study and outline directions for future research.

## 1. Introduction

Studies linked the effective use of information technology (IT) to productivity benefits in a wide variety of markets and industries, including healthcare [1–3]. Modern hospitals use IT to transform healthcare delivery processes, and thereby try to improve clinical quality, service efficiency, and patient satisfaction and reduce costs [3–7]. Hospitals do so because healthcare is a critical social and economic component of modern society, and the adoption of groundbreaking IT is essential to its success [7–13]. We mention a particular system that facilitates physicians and doctors on a day-to-day basis, namely, a clinical decision support system (CDSS). CDSSs are developed to enhance the process of clinical decision making and provide clinicians with several modes of decision support (e.g., alerts, reminders, advice) for active care issues according to specific clinical guidelines [14, 15]. CDSSs are known for their contribution to clinical performance, e.g., the reduction of medication error rates [16], improvements in antibiotic use, cost reduction [17, 18], improvements in drug dosing and preventive care [19], and enhancements of quality [18]. Studies attribute many benefits to the use of CDSSs within hospitals. However, empirical evidence for clinical, economic, workload, and efficiency outcomes remains sparse [20]. Thus, despite the substantial potential for clinical, operational benefits and performance gains from CDSSs, there have been limited studies on the antecedents and mechanisms underlying successful clinical decision support capability (CDSC) deployments in hospitals. Moreover, the focus of many investigations has narrowed the scope often to a specific patient and clinical outcomes (and also often on specific diseases [15]), and not so much those (IT-driven) aspects that enable this critical capability. Following Goh et al. [21], we now argue that it is paramount to study these aspects in detail so that this could contribute to our general understanding of IT implementations in hospitals, CDSSs in particular.

As hospitals more and more adopt health IT, decision-makers should increase attention to justification and evaluation of investments in IT. IT evaluations and targeted IT investments are becoming even more critical considering that extant literature has put forward that it is not uncommon that IT can impede organizational performance gains [1, 22–24]. This “paradox” drives a question of central importance, i.e., how hospital enterprises can leverage their IT investments to facilitate and enable clinical decision support?

Within this study, we draw upon the resource-based view of the firm (RBV) [25, 26] to position the deployment and usage of IT as a unique and difficult-to-imitate resource of value for hospitals [27]. Following the RBV—widely acknowledged as a prominent and influential theoretical framework for IT business value research—we argue that IT is a strategic source of value for hospitals. However, this source of value cannot operate on its own. Instead, recent insights suggest that this value is a result of the process of leveraging complementary IT resources [27, 28].

Given the above, the primary objective of this paper is to empirically examine the degree to which specific IT-enabled capabilities, i.e., (1) health information exchange (HIE) and (2) hospitals' information capability (IC) drive enhanced CDSC within hospitals. Following Bharadwaj [29], we develop the concept of IT as a “capability” and want to examine if IC and HIE collectively impact hospital's CDSC. IC is as a hospital's ability to leverage its data and information resources and clinical assets to support decision making within the hospital enterprise. HIE concerns the degree to which hospitals enable to share and exchange health and clinical data, e.g., laboratory results, physician documentation, and medication lists across the organizations' boundaries [30].

Therefore, we guide our research through the following three specific research questions:

1. To what extent does HIE influence the formation of an IC within hospitals?
2. To what extent does HIE influence the formation of CDSC within hospitals?
3. To what extent does IC mediate the relationship between HIE and CDSC within hospitals?

For this research, we follow a deductive approach to reach our study objectives. In doing so, we base claims in the RBV theory, focus on transparent research design and execution, and the development of logical arguments to substantiate our claims. We structured the remainder of this study as follows. First, we briefly review the literature on IT resources and the RBV of the firm. These two aspects form the theoretical foundation of this work. Next, we propose the research model and the three associated hypotheses. In the following sections, we outline the methods and present the most important results. Finally, we conclude this work and highlight the implications of our findings. In this current work, we also identify study limitations and present various avenues for future research.

## 2. IT Resources and the Resource-Based View of the Firm

Heavily resting on strategic management literature, the acknowledged RBV is a contemporary theory that explains how organizations (private and public) can achieve and sustain a competitive advantage as a result of the (IT) resources they own or have under their control [25, 26]. This theory seems a particularly interesting “lens” for hospitals that need to leverage their IT resources to reduce operating costs, enhance service quality, and improve patient care. Within the RBV, organizational resources represent the essential input of the processes, while capabilities represent the capacity to deploy these particular (IT) resources, aiming to achieve a particular goal (e.g., improved productivity, profits, quality improvement, and enhanced care). Using the RBV lens, scholarship postulates that the targeted use of IT assets and resources can be a differentiating force within a firm [27, 31]. Notwithstanding, the potential of IT resources and IT-enabled capabilities can only be achieved if they are sufficiently leveraged to improve the efficiency and effectiveness of business processes and core competencies [27, 32, 33]. These insights radically extend the early insights on IT investments and an apparent lack of measured productivity enhancements.

A growing body of scholarship now acknowledges that organizations should identify those capabilities that IT should target to enable or strengthen, to address rapidly changing business environments [34–36]. This crucial insight particularly suits the hospital environment and extends the core idea behind the RBV that organizations should foster business processes that are inimitable and leverage the core IT resources [37].

Recent studies acknowledge that the process of leveraging complementary IT resources is a crucial source of IT business value creation [27, 28]. Even so in healthcare, as research argues that the development of digital capabilities in clinical practice requires a high level of sophistication regarding resource allocation [38]. Failure to invest in IT resources—that are valuable, rare, inimitable, and nonsubstitutable—may cause the collapse of the value of resources and capabilities, making it necessary to place equal importance to each [39, 40].

Drawing on the RBV, we synthesize from the extant literature that hospitals should target those complementary IT-enabled capabilities that enhance CDSC within the hospital enterprise.

## 3. Research Model and Hypotheses

**Figure 1:**
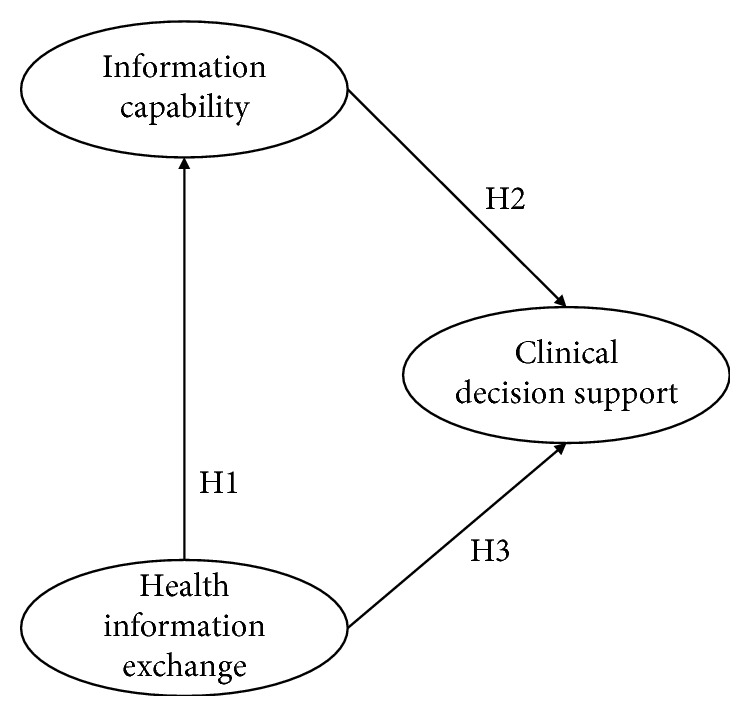
Research model showing the hypothesized relationships.

Our research model contains three constructs with associated hypotheses.  Figure 1 shows the entire research model that we will empirically validate. First, our research model concerns the relationship between HIE and IC (hypothesis one). The second element of our model concerns the relationship between the mediating construct (IC) and our dependent construct, i.e., CDSC within hospitals (hypotheses two). Finally, our model also contains a direct effect of HIE on hospitals' CDSC (hypothesis three). We will now elaborate on each of these three hypotheses.

### 3.1. Hypothesis Related to HIE and IC (H1)

Knowledge and data-intensive capabilities do not merely depend on organizations' direct interface with the external environment, but actually, also on the transfer of knowledge across and within the organization [41–43]. HIE is a data-intensive capability and allows hospitals to securely exchange and use health data and information in real time and offers potential to improve healthcare quality, lower operational costs, and reduce medical errors [44]. Moreover, it provides hospitals with the opportunity to share and process information among doctors, patients, and other stakeholders within the hospital ecosystem. Information sharing is critical because information is the backbone of hospital operations and hence provides high-quality services to patients. Therefore, modern hospitals are now considering the adoption and use HIE as a source of IT business value [45, 46]. Mature IT within hospital enterprises provide patients with instantaneous information from anywhere and anyone [45, 47]. HIE—as a critical capability—can facilitate this. HIE thereby contributes to the process of integrating various sources of health information and data. This integration is essential because hospitals want to be able to capture a complete patient image. However, exchanging data to obtain a complete patient image alone is not enough. We can easily conceive that the exchanged information and patient data need to be exploited even further and targeted into another IT-enabled capability to create value. Vital patient data and information need to be viewed and be used in clinical practice by doctors and nurses. Hence, we define the following hypothesis:

**Hypothesis 1 .**

HIE has a positive impact on the IC within the hospital.

### 3.2. Hypothesis Related to CI, HIE, and CDS (H2 and H3)

Following Li et al. [48], we see an IC as an IT-enabled capability that allows hospitals to capture a complete patient's picture and their behavior. We foresee that this particular capability will enhance the CDSC. In the current research context, IC will provide clinicians with information about patients (i.e., who they are), what conditions they might have, or what patients previously have been diagnosed with (i.e., what), where patients are from, and how they came at this particular place. It is well known that hospital operations depend heavily on the acquisition, exchange, analyses, and utilization of health and administrative information within and across the above organizational boundaries [49, 50]. So, IC seems critical in clinical care where decision making is highly dependent on accurate information and its usage. Given the above, it seems likely that it is crucial for hospitals to develop a high level of IC, which subsequently can enhance the process of clinical decision making.

**Hypothesis 2 .**

IC has a positive impact on CDSC within the hospital.

Previous studies have shown that HIE allows hospitals to efficaciously exchange and share clinical information across the organizations' boundaries [30]. Perhaps most importantly, HIE can enhance operational efficiencies and provide hospitals with the ability to push clinical data from one provider to another [44, 51]. This ability to effectively share critical care information and patient data (from various departments and other hospitals) is crucial for a patient's clinical pathway. In many scenarios and (even emergency) cases, critical patient information is vital to the patient's well-being and even survival.

HIE reduces possible barriers of distance, enhances access to critical clinical information, and makes valuable contribution to integrated care [52]. There are, apparently, some conditioning factors that hospitals need to take into account in practice. These include the limitation of the amount of redundant information and the avoidance of information and cognitive overload for doctors [46]. In any case, HIE contributes to the primary data and information need which is essential in patient management, safety, and clinical decision making [53, 54].

Hospitals should exchange data electronically to allow medical staff to access patients' clinical data sources across various sites of care to enhance their decision-making processes [55]. We, therefore, expect that HIE will provide value-added services for doctors. Hence, we define the following hypothesis:

**Hypothesis 3 .**

HIE has a positive impact on CDSC within the hospital.

## 4. Materials and Methods

### 4.1. Data Collection and Sample

To test the theorized relationships and three hypotheses of our research model, it is essential that we obtain a significant amount of cross-sectional data from hospitals. For this, we found a unique and large-scale dataset, the European Hospital Survey: Benchmarking Deployment of eHealth Services (2012-2013). This particular cross-sectional dataset is distributed by the European Commission (the survey is accessible through: https://ec.europa.eu/digital-single-market/en/news/european-hospital-survey-benchmarking-deployment-ehealth-services-2012-2013), as e-health is on the policy agenda of the European Commission for more than a decade. The objective of this particular study was to benchmark the level of e-health adoption and use in acute hospitals across 30 countries in Europe. The research approach and associated survey were based on results described in a report by Deloitte/Ipsos (2011) (Deloitte/Ipsos, 2011, eHealth Benchmarking (Phase III): final report for the European Commission, Brussels). The European Hospital Survey intentionally focused on European acute hospitals to guarantee coherence and comparability with the previous investigations.

The survey categories and blocks and covered a wide range of aspects from (a) IT infrastructure, (b) IT applications, (c) health information exchange, (d) security/privacy issues, and (f) IT functionalities. The survey was first piloted to improve the quality further. The survey targeted the Chief Information Officers (CIOs) of the acute hospitals given their broad knowledge of all these particular (technical) aspects. The CIO was not always available. Thus, depending on availability, interviews either started immediately or they were rescheduled to a future date.

Interview on average lasted approximately 45 minutes. Shortly after a pilot phase in October 2012, the data collection commenced and lasted until February 2013. The research team used computer-aided telephone interviewing (CATI) with native-speaking interviewers. Thus, all performed interviews were conducted in one of the official languages of the respective countries. Next to CATI, also an online survey was provided to improve response rates.

The 2012/2013 benchmarking study contains roughly 1.800 European hospitals. This amount is a result of reaching out to 26,550 healthcare establishments. Of these European hospitals, 5,424 qualified as acute care hospitals. In total, 1,753 hospitals completed an interview. The Benchmark was carried out by PwC EU Services, in cooperation with Global Data Collection Company (GDCC). GDCC collected the survey data.

Following our current research scope and used constructs in this research (Section 4.2), we improved the data quality by conservatively removing 1033 cases with lots of missing data entries. For data consistency and comparability, we additionally removed private and private not for profit hospitals (*N*=367) and University hospitals (*N*=196) from our sample. The organizational structure, processes, and financing mechanisms (also for IT) can differ considerably with the public hospitals. Our final dataset includes 720 hospitals across 29 countries in Europe.

We grouped our sample by firm size-class (using the number of beds), 13% large (750+ beds), 27% medium (251–750 beds), 51% small (101–250 beds), and 9% micro (less than 100 beds). We also clustered the hospitals across the 29 countries that are present in this sample (Table 1).

To control, *ex-post*, for common method bias (CMB), we performed Harman's single factor test using SPSS v24 on the included constructs in our current study. Hence, we loaded all construct variables on to a single construct in an exploratory factor analysis (EFA). As we could not find a single factor that attributes to the majority of the variance, we conclude that our sample is not affected by CMB [56].

### 4.2. Survey Items and Construct Measurements

HIE is a data-intensive capability that refers to a hospital's capability to securely exchange and use health data and information in real time. For this research, we devised a set of twelve survey items from the European Hospital Survey to operationalize HIE, including interaction with patients, appointments, transfer prescriptions, and exchange patient medication.  Table 2 shows all measurements for this particular construct and the descriptive statistics.

Our second construct concerns IC containing 17 survey items from the large-scale cross-sectional dataset including medication list, lab and radiology results, medical history, allergies, immunizations, and ordered tests.

IC is a critical care capability that in practice allows hospitals to capture a complete picture of a patient based on obtained health that is so important for decision-making processes within the hospital enterprise.  Table 3 shows all items for this particular construct and associated descriptive statistics.

Finally, we measure CDSC using six survey items as a representation of hospitals' capability to enhance the process of clinical decision making and provide clinicians with several modes of decision support.  Table 4 shows all survey items and descriptive statistics for CDSC.

All the above survey items were measured on a Likert scale from 1 to 5, where 1 denotes “not in place” and 5 denotes “fully implemented across all units.”

### 4.3. Model Specification and Validation

This study employs the second generation structural equation modeling (SEM) technique partial least squares (PLS) analyses. We do so, to validate the measurement model and examine the structural model to test the associated hypothesized relationships [57]. Our research model contains first-order latent constructs that are reflective of nature. Hence, the manifest variables are affected by the latent variables [58]. Also, the meaning of the construct does not change if items (with low loadings) are removed from the measurement model. The items reflect and depict the construct. We estimated the parameters of our research model using SmartPLS version 3.2.7. [59], which is an SEM application using PLS. We use this application to test both the inner (measurement) and the outer (structural) model. Also, we employed a nonparametric bootstrapping procedure to compute the level of the significance of the regression coefficients. We used 5000 replications to obtain stable results and to interpret their significance. The 720 hospitals in our sample far exceed all minimum requirements concerning the measurement and structural model [60, 61].

## 5. Results and Discussion

### 5.1. Analyses of the Measurement Model

To analyze the measurement model, we assessed the psychometric properties for all the first-order constructs on satisfactory levels of validity and reliability. We subjected our constructs to internal consistency reliability test, convergent validity test, and discriminant validity test through SmartPLS [59]. At the construct level, we checked internal consistency reliability using Cronbach's alpha (CA). Hence, we examined if all CA values were above the threshold of 0.70 [61, 62]. Complementary to CA, we computed the composite reliability (CR) values for each construct as this measure takes into account the loadings of the manifest variables [57]. Typically, CR values should be between 0.60 and 0.90, as is the case in our research (Table 5). Also, we assessed the construct-to-item loadings. Following [63], we removed all manifest indicators with a loading of less than 0.6 from our model. In total, we removed seven indicators from the HIE construct (i.e., nos. 1, 2, 3, 4, 10, 11, and 12). Finally, we removed eight indicators from the IC construct (i.e., nos. 1, 2, 3, 4, 5, 10, 12, and 17).

Researchers should also evaluate the measurement model by their convergent and discriminant validity [57, 61]. We assessed the convergent validity by examining if the average variance extracted (AVE) is above the lower limit of 0.50 [64]. All AVE values are above the minimum threshold. Next, we assessed discriminant validity through three different, but related tests. The first method examines if the cross loadings (i.e., correlation) on other constructs are less than the outer loading on the associated construct [65]. Second, we assessed the Fornell-Larcker criterion. Hence, we investigated if the square root of the AVEs of all constructs is larger than the cross correlation [66]. All correlations among all constructs were below the threshold (0.70) [61]. Third, and finally, we employed the heterotrait-monotrait (HTMT) ratio of correlations approach by Henseler et al. [67]. The HTMT is calculated based on the mean of the correlations of indicators across constructs measuring different constructs, relative to the average correlations of indicators within the same construct. All HTMT values showed acceptable outcomes far below the conservative 0.85 upper bound (Table 5). In summary, the outcomes (Table 6) suggest that the first-order reflective measures are valid and reliable. We can now evaluate the structural model.

### 5.2. Model Fit and Predictive Relevance Analyses

Before assessing the structural model and associated hypotheses, we checked the model by assessing the model fit (the included model fit indices should be interpreted with caution as model fit is not an established PLS-SEM evaluation criterion). Studies proposed the standardized root mean square residual (SRMR) as a model fit measure that calculates the difference between the observed correlation and the model implied correlation matrix [57, 68]. Our obtained 0.057 is far below the conservative 0.08 that is proposed by [68]. We additionally assessed a relatively new RMS_*theta*_ value that calculates the degree to which the measurement model residuals correlate (Lohmöller, 1989). Also, this measure shows that our mediated model is well fitting as it approximates 0 [69].

Finally, we also assessed the model's predictive relevance calculating the *Q*^2^ of our endogenous constructs (i.e., using the Stone–Geisser test). In doing so, we assess the quality of each structural equation measured by the cross-validated redundancy and communality index using blindfolding [70]. *Q*^2^ values > 0 imply the model's predictive relevance; values less than 0 suggest the model's lack of predictive relevance. In this study, all *Q*^2^ values for both CDS (i.e., 0.130) and IC (i.e., 0.071) are above the threshold value of zero, thereby indicating the overall model's predictive relevance. We can now estimate the structural model and the hypothesized relationship among the model's constructs to test the hypotheses.

### 5.3. Structural Model Analyses

**Figure 2:**
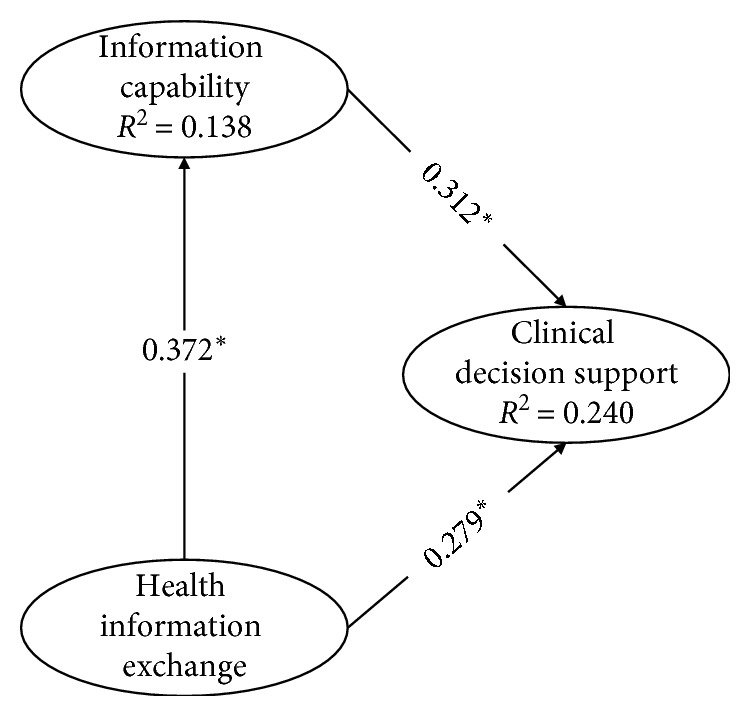
Estimated causal relationships of the structural model. Note: ^*∗*^*p* < 0.0001.

We summarize the structural model from the PLS analyses in Figure 2. It presents both the explained variance of endogenous variables (*R*^2^) and the standardized path coefficients (*β*). As discussed earlier, we obtained the significance of estimates (*t*-statistics) by performing a bootstrap analysis with 500 resamples. Outcomes of these analyses support all three hypotheses. HIE is significantly related to IC (*β* = 0.372; *t* = 10.496; *p* < 0.0001). In turn, IC is positively linked with CDS (*β* = 0.312; *t* = 7.744; *p* < 0.0001). In addition, HIE has a positive and significant influence on CDS (*β* = 0.279; *t* = 6.960; *p* < 0.0001).

Our structural model explains 13.8% of variance for IC (*R*^2^ = 0.138) and 24% for CDSC (*R*^2^ = 0.240). These particular coefficients of determination represent moderate to substantial predictive power [57].

To specifically address the question to what extent IC mediates the relationship between HIE and CDSC within hospitals, we followed the guidelines by Hair et al. [57] for mediation analysis procedures. Thus, we first addressed the significance of the indirect effects. Following the above analyses, we found support for the hypothesized mediating relationship. Also, the direct effect (HIE ⟶ CDSC) is significant. To additionally check whether this indirect effect (thus the product of direct and the indirect effect; 0.372 × 0.312 = 0.116) is significant, we employed a bootstrapping approach using a nonparametric resampling procedure that imposes no assumptions on the normality of the sampling distribution [57]. We found that this indirect effect is significant (*t* = 6.578; *p* < 0.0001). The direct effect and the indirect effect are both positive and significant. We, therefore, conclude that there is a complementary partial mediating relationship.

Next, to examining the *R*^2^ of the endogenous constructs, we also evaluate effect sizes, *f*^2^. With effect sizes, we can determine the specific contribution of particular exogenous constructs to an endogenous latent constructs *R*^2^. Hence, these outcomes demonstrate moderate effect sizes.  Table 7 summarizes the outcomes of the structural model analyses.

In addition to the above measurement and structural model analyses, we controlled for possible unobserved heterogeneity in our dataset by employing the finite mixture (FIMIX) PLS procedures [71]. Hence, we sequentially segmented the dataset into various segments (s2–s4)—taking minimum sample size requirements into account of 100 for reliable estimation of the model parameters [72]—to identify whether there are possible conditioning factors that currently are not incorporated into our research model and analyses. These segments then might explain observed differences across various groups of hospitals [73]. Outcomes of the FIMIX analyses suggest that higher levels of explained variance can be achieved for some homogeneous subgroups. It could well be the case that the region of the hospital, type, or other organizational and environmental aspects play a crucial role here. An extensive *ex-post* FIMIX-PLS analysis is beyond the scope of this current paper.

### 5.4. Discussion, Practical Implications, and Limitations

Clinical decision support within hospitals has received a growing amount of attention in scientific literature. However, despite the substantial potential for clinical, operational benefits and performance gains from CDS, empirical research investigating the core antecedents and mechanisms underlying successful CDSC within the hospital enterprise remains modest. Grounded in the RBV, this study examined the structural relationships among HIE, IC, and CDSC in European hospitals using cross-sectional survey data from the European Hospital Survey. The outcomes of this research show that HIE positively influences a hospital's IC so that hospitals can strengthen its ability to capture a complete patient image for clinical operations. Furthermore, our results demonstrate the IC, in turn, positively influences hospitals' CDSC. It thus seems that IC is a crucial IT-enabled capability to enhance the process of clinical decision making. Finally, we also uncovered a positive direct impact of HIE on CDSC. This direct effect shows the substantial role of HIE in practice, supporting the claim that HIE enhances access to critical clinical information and makes valuable contribution to decision-making processes of doctors, patient management, and integrated care [52–54]. After running various statistical analyses, we confirmed the complementary partial mediating relationship within our research model.

These insights provide support for the appropriateness of the RBV “lens.” The current study also has some interesting evidence-based implications for practice as the outcomes suggest that managers and decision-makers should target the complementary IT-enabled capabilities HIE and IC simultaneously to enhance CDSC within the hospital enterprise. These results are significant because they contribute to our understanding of how to leverage complementary IT resources and capabilities as a strategic source of value and value-added services within hospital enterprises. It is well known that IT implementations in healthcare might be hindered by various organizational and technical barriers [74, 75]. Therefore, it is essential to know which IT-enabled capabilities managers should strengthen so that IT can be beneficial to improve the health of individuals and the performance of doctors. In practice, we see that hospital decision-makers struggle on a daily basis with the adaption, use, and targeted investments of IT and digital technologies. Our study outcomes thus support hospital managers (e.g., CIOs) in coping with a multitude of developments and enduring challenges while simultaneously leveraging current IT, competences, and capabilities for optimal contributions to CDS.

Like all research, some limitations constrain our study, so the outcomes need to be interpreted with caution. First, we currently only included two essential antecedent capabilities into our research model. In practice, other organizational capabilities and contextual aspect might condition CDSC. Future research may wish to investigate other conditions, configurational patterns, and antecedents of CDSC. Hence, scholars could benefit from comprehensive *ex-post* FIMIX-PLS and configurational analyses so that multiple group and (sub) segment comparisons can unfold new relevant insights. A second potential limitation is that we only focused on public hospitals. We believe this approach is justified. However, future research could also explore the differences between public and semiprivate hospital enterprises. Finally, this study does not provide the details that are necessary to commence an implementation or improvement project concerning CDSC, as this is beyond our current scope of the paper.

## 6. Conclusions

To conclude, it goes without saying that CDS is essential for hospitals. A mature CDS capability will provide hospitals with clinical, operational, and other performance benefits. To our current knowledge, our study is the first to empirically investigate the degree to which health HIE and a hospital's IC drive enhanced CDSC within hospitals. By considering the key antecedents and mechanisms through which CDSC can be foundationally be enhanced, we make a valuable contribution to the medical practice and the academic community. Our work also serves as a basis for future theoretical and applied health IT investigations.

## Figures and Tables

**Table 1:** Sample characteristics.

	Frequency	Percentage (%)
*Size (amount of beds)*		
Fewer than 101 beds	96	9
Between 101 and 250 beds	193	51
Between 251 and 750 beds	365	27
More than 750 beds	66	13

*Country*		
Austria	17	2.4
Belgium	14	1.9
Bulgaria	17	2.4
Croatia (local name: Hrvatska)	5	0.7
Czech Republic	16	2.2
Denmark	5	0.7
Estonia	11	1.5
Finland	21	2.9
France	182	25.3
Germany	45	6.3
Greece	37	5.1
Hungary	29	4.0
Iceland	8	1.1
Ireland	3	0.4
Italy	80	11.1
Latvia	8	1.1
Lithuania	10	1.4
Luxembourg	1	0.1
Malta	2	0.3
Netherlands	17	2.4
Norway	1	0.1
Poland	49	6.8
Portugal	19	2.6
Romania	44	6.1
Slovakia (Slovak Republic)	18	2.5
Slovenia	2	0.3
Spain	29	4.0
Sweden	14	1.9
United Kingdom	16	2.2

**Table 2:** Health information exchange survey items and descriptive statistics.

HIE construct items	Mean	SD
(1) Interact with patients by e-mail about health-related issues	4.71	0.61
(2) Make appointments at other providers on patients' behalf	4.63	0.66
(3) Send/receive a referral and discharge letters	4.49	0.74
(4) Transfer prescriptions to pharmacists	4.60	0.66
(5) Exchange patient data with other healthcare providers and professionals	4.42	0.77
(6) Receive laboratory reports	4.60	0.64
(7) Receive/send laboratory reports and share them with healthcare professionals/providers	4.49	0.75
(8) Exchange patient medication lists with other healthcare professionals/providers	4.55	0.72
(9) Exchange radiology reports with other healthcare professionals/providers	4.47	0.76
(10) Exchange medical patient data with any healthcare provider in other countries	4.88	0.39
(11) Certify sick leaves	4.65	0.69
(12) Certify disabilities	4.81	0.48

**Table 3:** Information capability survey items and descriptive statistics.

IC construct items	Mean	SD
(1) Medication list	4.51	0.68
(2) Prescription list	4.43	0.70
(3) Lab test results	4.81	0.47
(4) Radiology test results (reports)	4.74	0.52
(5) Radiology test results (images)	4.67	0.56
(6) Problem list/diagnoses	4.57	0.65
(7) Reason for encounter	4.54	0.63
(8) Allergies	4.52	0.69
(9) Encounter notes, clinical notes	4.52	0.68
(10) Immunizations	4.41	0.82
(11) Vital signs	4.44	0.73
(12) Patient demographics	4.75	0.51
(13) Symptoms (reported by the patient)	4.56	0.66
(14) Medical history	4.53	0.67
(15) Ordered tests	4.54	0.69
(16) Disease management or care plans (e.g., diabetes)	4.43	0.72
(17) Finance/billing information	4.72	0.58

**Table 4:** Clinical decision support capability survey items and descriptive statistics.

CDS construct items	Mean	SD
(1) Clinical guidelines and best practices (e.g., alerts, prompts)	4.29	0.83
(2) Drug-drug interactions	4.37	0.79
(3) Drug-allergy alerts	4.37	0.80
(4) Drug-lab interactions	4.30	0.84
(5) Contraindications (e.g., based on age, gender, pregnancy status)	4.32	0.82
(6) Alerts to a critical laboratory value	4.57	0.62

**Table 5:** The assessment of heterotrait-monotrait ratio of correlations (HTMT).

	(1)	(2)	(3)
(1) CDS	**—**		
(2) HIDE	0.473	**—**	
(3) Information capability	0.469	0.433	**—**

**Table 6:** Assessment of convergent and discriminant validity of reflective constructs.

	(1)	(2)	(3)
(1) CDS	**0.767**		
(2) HIDE	0.395	**0.751**	
(3) Information capability	0.416	0.372	**0.751**

AVE	0.589	0.564	0.564
Cronbach's alpha	0.856	0.804	0.902
CR	0.894	0.865	0.920

**Table 7:** Summary of the three hypotheses and outcomes of the structural model analyses.

Structural model path	Effect size (*f*^2^)	Bias-corrected confidence interval	Significant	Conclusion
HIE ⟶ IC	0.161	CI (0.302–0.444)	Yes	H1 supported
IC ⟶ CDSC	0.111	CI (0.242–0.380)	Yes	H2 supported
HIE ⟶ CDSC (direct)	0.088	CI (0.188–0.347)	Yes	H3 supported
HIE ⟶ CDSC (indirect)	—	CI (0.085–0.158)		

Note: CI = confidence interval (lower bound, 2.5%; upper bound, 97.5%).

## Data Availability

The data from the “European Hospital Survey: Benchmarking Deployment of eHealth Services (2012-2013)” are publicly available on the European Commission website.
